# Limb bone robusticity is coupled with mass distribution in terrestrial tetrapods

**DOI:** 10.1098/rsos.251103

**Published:** 2025-09-10

**Authors:** Matthew Dempsey, Kai Allison, Samuel R. R. Cross, Susannah C. R. Maidment, Nicolás E. Campione, Karl T. Bates

**Affiliations:** ^1^ Department of Musculoskeletal & Ageing Science, University of Liverpool, Liverpool, UK; ^2^ Fossil Reptiles, Amphibians and Birds Section, Natural History Museum, London, UK; ^3^ Palaeoscience Research Centre, School of Environmental and Rural Science, University of New England, Armidale, New South Wales, Australia; ^4^ School of Geography, Earth and Environmental Sciences, University of Birmingham, Birmingham, UK

**Keywords:** limb bones, centre of mass, gait, posture, tetrapods, dinosaurs

## Abstract

The vertebrate body is a highly modular system within which evolutionary adaptation is expected to occur synchronously at a variety of hierarchical scales, from single tissue to whole organism. For example, the evolution of different body shapes, associated with disparate locomotor ecologies, will affect the loading regimes experienced by limbs, and may therefore be coupled with adaptations to limb bone morphology. However, such a relationship between body shape, limb loading and bone morphology has not been tested. Here, we find significant positive relationships between whole-body relative anteroposterior centre of mass and the robusticity of the humeral shaft relative to the femoral shaft across a disparate sample of tetrapods. As centre of mass shifts towards the shoulder, the humerus becomes proportionally more robust. However, the magnitude of this increased robusticity and the anatomical planes across which it occurs vary between tetrapod clades, reflecting the different limb loading regimes imposed by postural differences. These relationships illuminate the osteological adaptations associated with variation in mass distribution and limb posture, and provide a framework within which centres of mass in fossil tetrapods such as dinosaurs can be predicted, opening the door to large-scale studies of tetrapod centre of mass and body plan macroevolution.

## 1. Introduction

The body plans of tetrapods vary adaptively according to their different ecological niche occupations and the mechanical demands imposed by movement, with this variation underpinning many important adaptive radiations throughout their evolutionary history [1–7]. In terrestrial tetrapods, body shape reflects mass distribution across the body, and is therefore a major indicator of the mechanical loading regimes experienced by the limbs [8–11]. For example, in bipeds, static stability requires the foot to be placed below the centre of mass, constraining the hind limb flexion required during midstance and influencing the direction of the ground reaction force relative to each limb segment [12]. In quadrupeds, more anterior centres of mass result in the forelimbs experiencing a greater proportion of the ground reaction force, or contributing more prominently towards acceleration [8,10,11,13]. Tetrapod long bones are structured to equilibrate strain and avoid instability, with different limb bone morphologies reflecting the varied loading regimes experienced during stance and locomotion [14–17]. It may therefore be expected that the morphology of the load-bearing limb bones may be adaptively coupled to the equilibration of varied locomotor stresses associated with changes to body shape across terrestrial tetrapods.

**Figure 1.**
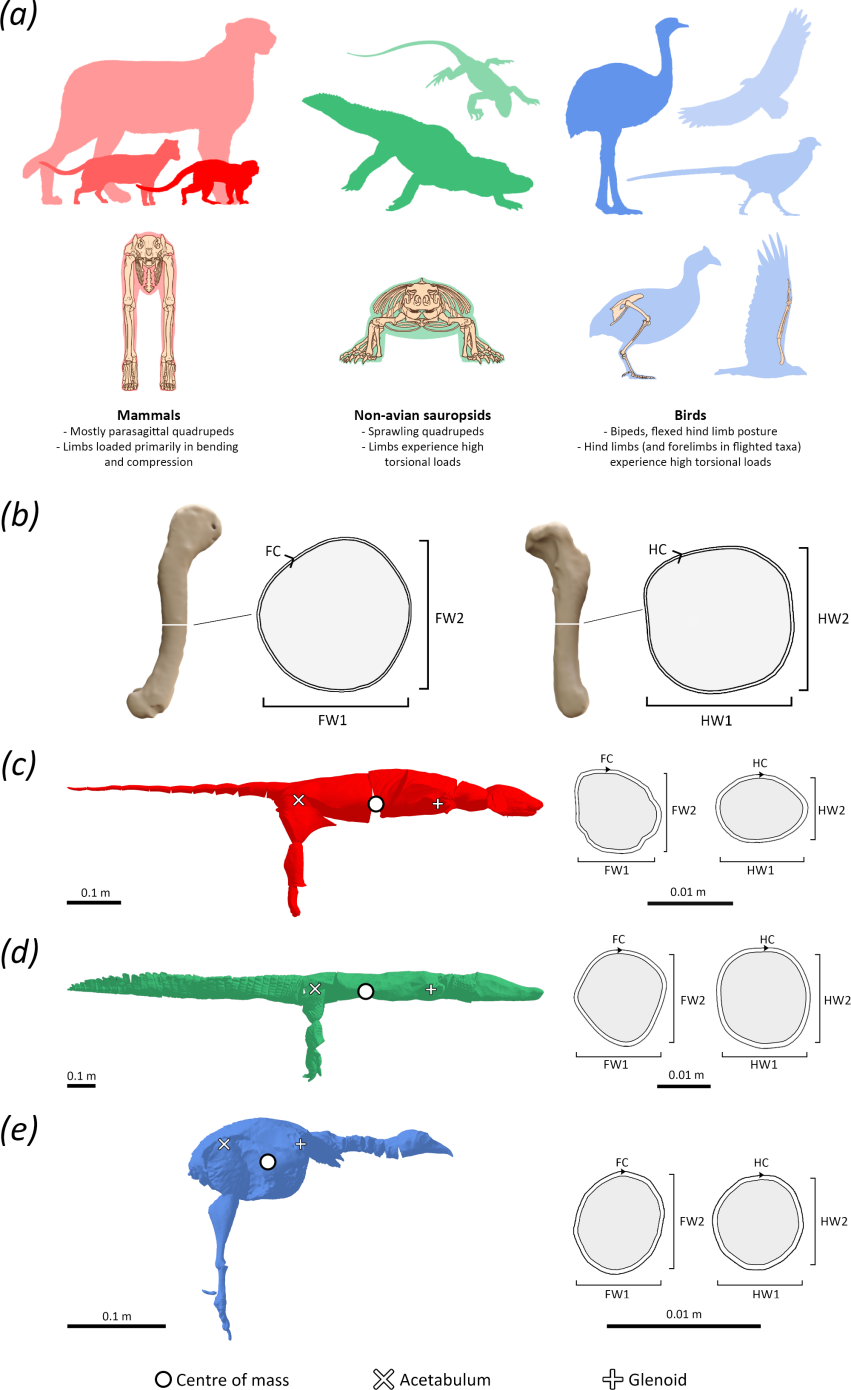
(*a*) Illustrations of the extant tetrapod groups studied herein, with general classifications of their gross posture and limb bone loading regimes. (*b*) Illustration of stylopodial shaft dimensions as measured from the femur (left) and humerus (right) of the Nile crocodile (*Crocodylus niloticus*). Volumetrically measured centres of mass and stylopodial shafts in section for a representative (*c*) mammal (masked palm civet, *Paguma larvata*), (*d*) non-avian sauropsid (Morelet’s crocodile, *Crocodilus moreletti*) and (*e*) bird (willow ptarmigan, *Lagopus lagopus*).

A considerable body of work on extant tetrapods has also highlighted that different loading regimes are seen in tetrapods with disparate limb postures. For example, the parasagittal limb bones of mammals are primarily loaded in bending and compression (figure 1*a*), whereas the sprawled limb bones of non-avian sauropsids and the flexed hind limbs of birds (as well as the forelimbs during flight) experience relatively high degrees of torsion (figure 1*a*), in addition to bending and compression [15–24]. In turn, these differences have been hypothesized to influence inter-clade differences in the scaling of limb bone morphology [15–17,21–25]. Potential developmental couplings between limb bone morphology and aspects of whole-body morphology, posture and locomotion are also reflected in the tetrapod fossil record. Diversifications in limb bone dimensions have been shown to characterize multiple major evolutionary transitions in body shape, body size and locomotor dynamics, such as the convergent secondary evolution of posturally diverse quadrupedal gaits in variably proportioned sauropodomorph and ornithischian dinosaurs [26,27], or the shift between sprawling and upright gaits in stem-mammals [28]. Limb bones also function as the fundamental weight-bearing components of terrestrial tetrapod bodies, and thus their dimensions are also well understood to be functionally coupled with body mass. Multiple studies have demonstrated that the cross-sectional shaft dimensions of limb bones are statistically robust proxies for body mass [25,29,30], with the summed circumferences of the humerus and femur of quadrupedal tetrapods showing a consistent relationship with body mass across multiple tetrapod clades [25].

Despite the well-understood associations between limb bone dimensions, body mass and postural disparity, the impact of the spatial distribution of said body mass (as a product of body shape) on limb bone dimensions, and how this may interplay with aspects of posture and locomotion, has yet to be broadly quantified across tetrapods. Given that the summed cross-sectional dimensions of the humerus and femur are strongly functionally coupled with body mass, it is intuitive to hypothesize that the humeral-to-femoral cross-sectional ratios will in turn reflect differences in the proportion of total body mass supported by each bone, as a product of varying whole-body centre of mass. If such a relationship can be identified, this would facilitate further interpretation of broader macroevolutionary patterns in limb and body dimensions across tetrapods both extant and extinct, and may prove particularly useful for interpreting body mass distribution and locomotor regimes in fossil taxa for which whole-body dimensions are unknown due to limited remains.

In this study, we provide the first quantitative examination of the relationships between properties of whole-body shape and relative fore-to-hind limb bone shaft size in tetrapods. As in previous studies [1,3,7,31] we focus on anteroposterior centre of mass as an effective proxy for body shape and proportions that allows for broad comparisons between a wide range of morphologically disparate taxa. We also discuss how postural factors that further affect the ways in which limb bones are loaded are also associated with disparate bone morphologies, and may therefore influence the trends via which limb bones scale with mass distribution in different tetrapod clades. The relationships examined in this study formed the basis of three overarching hypotheses:

*Hypothesis 1:* Based on previous observations of strong correlations between body mass and cross-sectional dimensions of weight-bearing limb bones in terrestrial tetrapods, we hypothesize that relative differences between forelimb and hind limb bone cross-sectional dimensions will also reflect the relative loading experienced by each, as determined by whole-body centre of mass. Specifically, we predict that increased cross-sectional robusticity of the humeral shaft relative to that of the femur is coupled with a more anterior centre of mass.

*Hypothesis 2:* The anatomical planes in which the stylopodial elements increase in relative cross-sectional robusticity vary between different tetrapod groups with disparate limb postures (figure 1*a*). We specifically propose that the relative position of the centre of mass in quadrupeds is primarily coupled with differences in relative cross-sectional limb shaft robusticity in the planes of elbow and knee extension, as a result of their limb bones being loaded in vertical bending and compression (e.g. [16,21]). This relationship is expected to be more constrained in mammals than in sprawling non-avian sauropsids, owing to the parasagittal gaits of the former minimizing torsional loading relative to the latter (e.g. [18,23]). The varied scaling relationships proposed by hypothesis 2 extend beyond quadrupeds—in birds, we hypothesize that changes to limb bone shaft dimensions would also be less constrained to specific anatomical planes, resulting from the torsion-dominated loading regimes experienced during flight and crouched gait (e.g. [16,24]).

*Hypothesis 3:* As centre of mass shifts anteriorly in tetrapods, humeral shaft robusticity increases relative to body mass as an adaptation to greater total stresses on the forelimbs. While hypotheses 1 and 2 aim to explore relationships between relative centre of mass and the dimensions of the forelimb shafts relative to the hind limb shafts, through hypothesis 3 we also seek to understand whether differences in body mass distribution are also coupled with the dimensions of the limb bone shafts relative to whole-body size.

Given that relative stylopodial dimensions can be reliably measured in the fossil record, support for hypotheses 1 and 2 would imply potential for their use as predictive proxies for centre of mass in both extant and extinct taxa, which would facilitate large-scale macroevolutionary analyses of body proportions and mass distribution in tetrapods. While still mechanistically important to consider, the relationships recovered in support of hypothesis 3 are not used predictively herein, as they rely on *a priori* knowledge of body mass, which must itself also be predicted in fossil taxa with varying certainty (see [31,32]). To explore the predictive capacity of hypotheses 1 and 2, we applied the recovered stylopodial index relationships to a dataset of non-avian dinosaur limb bone dimensions, representing most major clades and body plans. Dinosaurs were chosen over other extinct taxa due to the abundance of studies that have focused on estimating centres of mass from volumetric models, providing a comparative framework to examine and compare the predictive capacity of our new stylopodial equations in a macroevolutionary context.

## 2. Methods

### 2.1. Extant tetrapod data

Stylopodial dimensions and centres of mass were measured from three-dimensional (3D) skeleton and skin outline CT-based models of 57 extant tetrapods derived from previously segmented models [7,33] and newly segmented published CT datasets (Museum of Vertebrate Zoology, University of California, and Field Museum of Natural History, via MorphoSource). These included 10 mammals, 7 crocodylians, 9 lepidosaurs and 31 birds. Taxon and model details are listed in electronic supplementary material, SM2.

To obtain centres of mass, skin outline models were divided into segments and manipulated into a standardized reference pose using Blender v. 3.1 [34], following the protocol of Macaulay *et al*. [7], which was the source of the bird centres of mass. In this pose, the axial skeleton is aligned anteroposteriorly, the hind limbs dorsoventrally, and the forelimbs mediolaterally (figure 1*c*–*e*). Standardized reference poses such as these do not represent *in vivo* postures, but instead function as an anatomical comparative standard for disparate morphologies and body plans, and a consistent basis for volumetrically measuring centres of mass using established protocols [1,7,31,35]. Such standards are particularly useful in taxa for which postural kinematic data are unavailable, such as extinct taxa. To ensure that centres of mass measured from reference poses represent suitable proxies for *in vivo* values, we carried out a sensitivity analysis in which models of taxa with available kinematic data were reposed into approximate mid-stance postures, with the necks of the birds also reoriented into an approximated habitual elevated posture. These postural differences resulted in only minimal centre of mass displacement along the anteroposterior axis (see electronic supplementary material, SM1.1), supporting the ongoing use of the standardized reference pose.

While centre of mass is a 3D coordinate, whole body centre of mass is functionally a two-dimensional value that can be assumed to be mediolaterally constrained to the sagittal plane in a bilaterally symmetrical animal. Herein, as in many previous studies [1–4], we focus on the anteroposterior centre of mass, quantifying its relative displacement from the acetabulum using a non-dimensionalized value:


(2.1) $$Relative\;centre\;of\;mass\;=\frac{Anteroposterior\;displacement\;of\;centre\;of\;mass\;from\;the\;acetabulum\;}{Glenoacetabular\;distance}.$$

Dividing by glenoacetabular distance provides the most direct measure of the relative (i.e. size-corrected) proximity of the centre of mass to the forelimb versus the hind limb, which is most relevant to testing hypotheses about the relationship between mass distribution and the relative loading upon each limb. In addition to the relative centre of mass of the extant taxa, we measured several limb bone shaft dimensions (figure 1), including minimum humeral and femoral shaft circumferences (HC and FC, respectively), as well as humeral and femoral shaft widths measured at the minimum shaft circumferences parallel to the elbow and knee extensor planes (HW1 and FW1, respectively), and perpendicular to the elbow and knee extensor planes (HW2 and FW2, respectively). In a parasagittal limb, HW1 and FW1 are measured in the anteroposterior planes, and HW2 and FW2 are measured in the mediolateral planes, but as these planes vary in orientation relative to the body throughout the gait cycle of a sprawling limb, we have opted not to use those terms as strict identifiers. To test hypotheses 1 and 2, these stylopodial measurements were used to calculate three relative measures of humeral-to-femoral robusticity, hereafter referred to as stylopodial indices, including a circumference index and two width indices:


(2.2) $$C\;\left(circumference\right)\;index=\frac{HC}{FC},$$

(2.3) $$W1\;\left(width\;1\right)\;index=\frac{HW1}{FW1},$$

(2.4) $$W2\;\left(width\;2\right)\;index=\frac{HW2}{FW2}.$$

While hypotheses 1 and 2 specifically focus on cross-sectional measurements of robusticity, the relative lengths of limb bones may also vary alongside fore–aft mass distribution. We therefore calculated relative humeral length (HL) to femoral length (FL) indices for each taxon:


(2.5) $$L\;\left(length\right)\;index=\frac{HL}{FL}.$$

For hypothesis 3, each individual humeral and femoral shaft dimension was divided by total body mass to the power of one-third ($\sqrt[{3}]{mass}$). In the birds and most of the sauropsids, body masses were based on direct cadaver-measured values (see electronic supplementary material, SM2), whereas the body masses of the mammals and several smaller lepidosaurs were estimated from the volumetric models using heterogenous segment densities (see electronic supplementary material, SM1.2a, SM2). We also calculated mass-relative lengths for each humerus and femur, respectively ($length/\sqrt[{3}]{mass}$).

While our hypotheses focus primarily on relative humeral to femoral dimensions and their relationships with body mass, limb robusticity can also be quantified as the intrinsic robusticity of singular limb bones, expressed as a measure of their cross-sectional thickness or circumference relative to their length [36]. Therefore, we also calculated a set of ‘limb-intrinsic’ stylopodial indices in which HW1, HW2 and HC were each divided by HL, and FW1, FW2 and FC were each divided by FL.

Relationships between relative centre of mass and each stylopodial index and mass relative stylopodial measurement were investigated using ordinary least squares (OLS) regression in R version 4.2.2 [37]. Given that the limb shafts of extant tetrapod clades and/or functional groups are loaded in different ways [16,17], they may be expected to scale according to different exponents. As such, we evaluated linear relationships across specific subsets: mammals, non-avian sauropsids and birds. While lepidosaurs and crocodylians are only distantly related within sauropsids, they are grouped together herein based on assumptions of gross functional and mechanical similarity [16,38]. To compare overall model fits, we also computed the adjusted coefficient of determination (*R*^2^) and the sample-corrected Akaike information criterion (AiCc) for each OLS model. Data were log_10_-transformed prior to regression. While our hypothesized associations between stylopodial indices and relative centre of mass are primarily on the basis of function, we also assessed the possible effect of phylogeny on the recovered overall trends (or the lack thereof) by producing phylogenetic generalized least squares (PGLS) models using the nlme package version 3.1 [39] for R version 4.2.2 [37], using the same anatomical input data and subset variations as the OLS models, and trees derived from recent molecular phylogenies (see electronic supplementary material, SM1.2 and SM7), assuming a phylogenetic correlation structure based on a Brownian motion model of evolution. AiCc values were also computed for the PGLS models to compare their fit to the associated OLS models. Due to their large femora, small forelimbs and posterior centres of mass compared with other birds, ratites considerably expand the total variance of the bird dataset and thus have the potential to strongly skew any recovered relationships—we therefore produced a subsequent alternative version of each bird regression that excluded them. Residual boxplots were also used to identify possible outliers from the initial regression models. To avoid over-filtering of relatively small subsets, which may be sensitive to boxplot quartile calculation methods, taxa were only marked as outliers for subsequent exclusion if recovered as such by both exclusive and inclusive median quartile calculations. To assess the predictive potential of the models derived to evaluate hypotheses 1 and 2, the slope equations were applied to the stylopodial indices. Given that relative centre of mass is a non-dimensional value between zero and one, the overall prediction accuracy of each equation was assessed as the mean absolute difference between the volumetric measurements and stylopodial predictions.

### 2.2. Dinosaur stylopodial data and centre of mass predictions

Statistically significant extant tetrapod OLS stylopodial index regression equations (slope *p* < 0.05) were applied to the equivalent stylopodial indices in a selection of non-avian dinosaurs to predict their relative centres of mass. These dinosaur stylopodial indices were primarily derived from previous data (see electronic supplementary material, SM2), and sample the majority of non-avian dinosaur body plans and clades. To provide a framework within which stylopodial centre of mass predictions could be compared with other established approaches, we only determined stylopodial indices in taxa for which previous centre of mass estimates based on volumetric reconstructions were also available, primarily from Bishop *et al*. [4] and references therein, with additional data provided by Donald Henderson. Full details of each model are outlined in electronic supplementary material, SM2. Due to incomplete data, not every non-avian dinosaur in the dataset could be sampled for every stylopodial index. Following the same approach to the calculation of mean error in the extant regression models, overall variation between relative centre of mass estimates derived from extant regression equations and previous volumetric dinosaur models was quantified by calculating their mean absolute difference for each major clade (Theropoda, Sauropodomorpha and Ornithischia, respectively). To further assess overall congruence between our extant-based stylopodial estimates and previous volumetric estimates of centre of mass, each set of estimates was compared to the volumetric models using Spearman’s rank order correlation tests in R version 4.2.2 [37]. Importantly, this allowed us to assess whether relative differences and overall trends in centre of mass between taxa were retained between methods, irrespective of quantitative differences in individual estimates. To further assess whether recovered macroevolutionary trajectories in centre of mass were consistent between methods, we also compared ancestral state reconstructions of centre of mass at the internal nodes of the dinosaur tree based on both the stylopodial and volumetric centre of mass estimates. The dinosaur tree was derived from a modified version of the time-scaled tree from Bishop *et al*. [4], trimmed to the taxa used in each analysis (electronic supplementary material, SM7). Ancestral state reconstructions were generated using the FastAnc function of the Phytools package version 2.0-4 [40,41] for R version 4.2.2 [37].

An alternative set of linear models were generated in which the non-avian dinosaur stylopodial indices were regressed against their corresponding volumetric relative centre of mass estimates. Assuming that the previous volumetric reconstructions are approximately accurate (but see §4.4), this approach allowed us to assess unique non-avian dinosaur-specific relationships that may not have been captured by trends defined from extant tetrapod morphologies. As with the extant linear regressions, relationships were explored separately across major groups (in this case, theropods, sauropodomorphs and ornithischians) using both OLS and PGLS, with the latter using a Brownian correlation structure. Due to uneven sampling between indices, AiCc values can only be used to compare OLS and PGLS models for a given index, and not cross-index comparisons.

While the previous volumetric estimates of dinosaur centre of mass were produced using generally similar modelling methods (or made use of systematic correction factors to ensure greater comparability, see [4]), a certain degree of subjectivity regarding overall soft tissue dimensions remains inherent to such methods [3,7,31,32,35] (see also §4.4). To investigate the potential effects of this subjectivity on comparisons between volumetric and stylopodial index-based estimates, we repeated our sauropodomorph linear regressions and ancestral state comparisons using alternative sauropodomorph centres of mass derived from a set of recent methodologically distinct volumetric models that included most of the same taxa. Specifically, these values were the ‘preferred allometric’ and ‘preferred isometric’ models of Dempsey *et al*. [31], each of which utilized different scaling approaches based on relationships between minimal skeletal convex hulls and soft tissue volumes in extant sauropsids [7].

## 3. Results

### 3.1. Coupling between stylopodial shaft robusticity and centre of mass in extant tetrapods

Complete breakdowns of the extant linear regressions are provided in electronic supplementary material, SM3, with overall trends summarized here.

**Figure 2.**
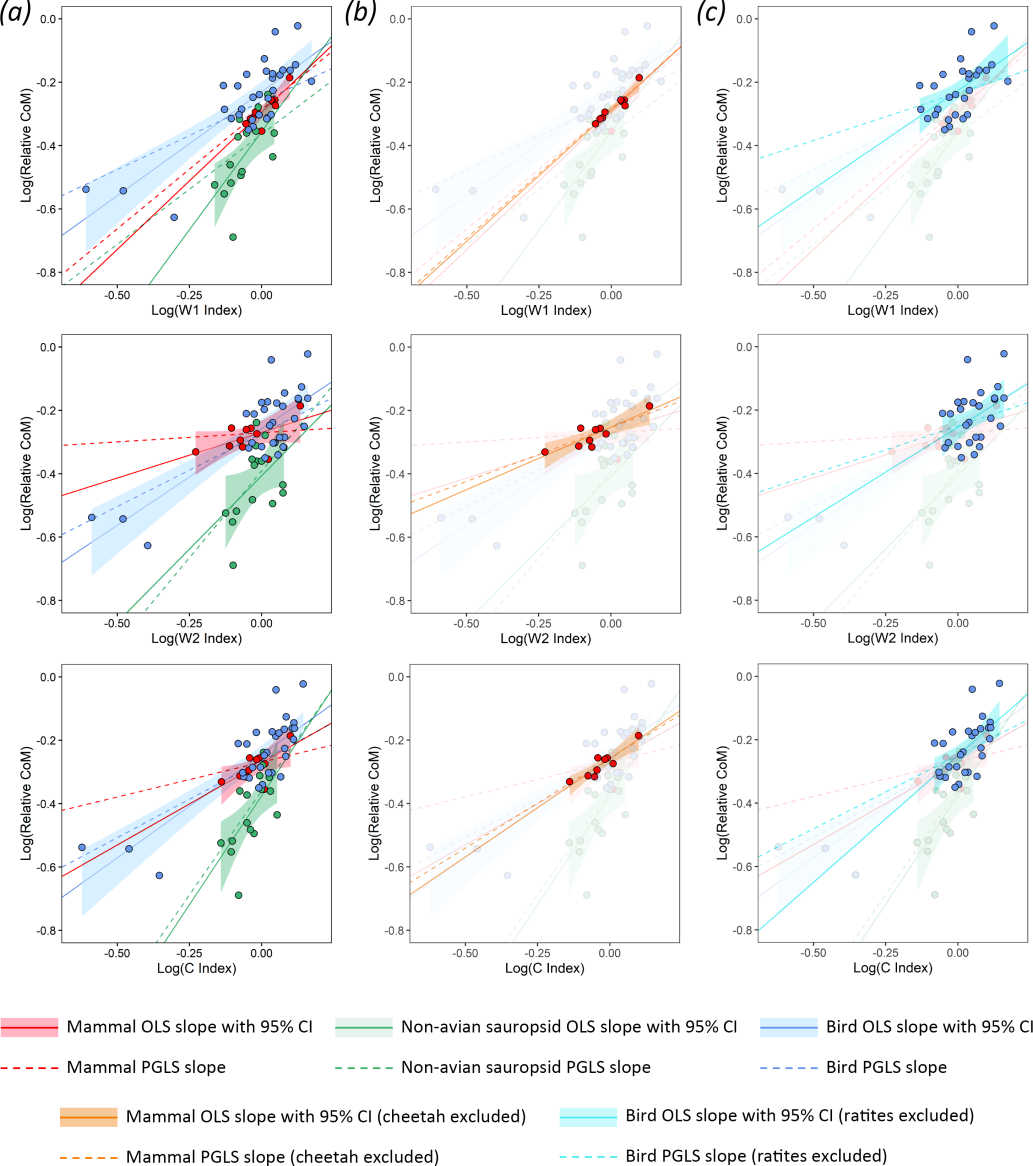
Scatter plots showing the results of the relative centre of mass–stylopodial cross-sectional index regressions in extant tetrapods. Indices include the W1, W2 and C indices, as described in the text. Plot columns represent (*a*) all taxa, (*b*) alternative mammal models with *Acinonyx jubatus* removed and (*c*) alternative bird models with ratites removed.

**Figure 3.**
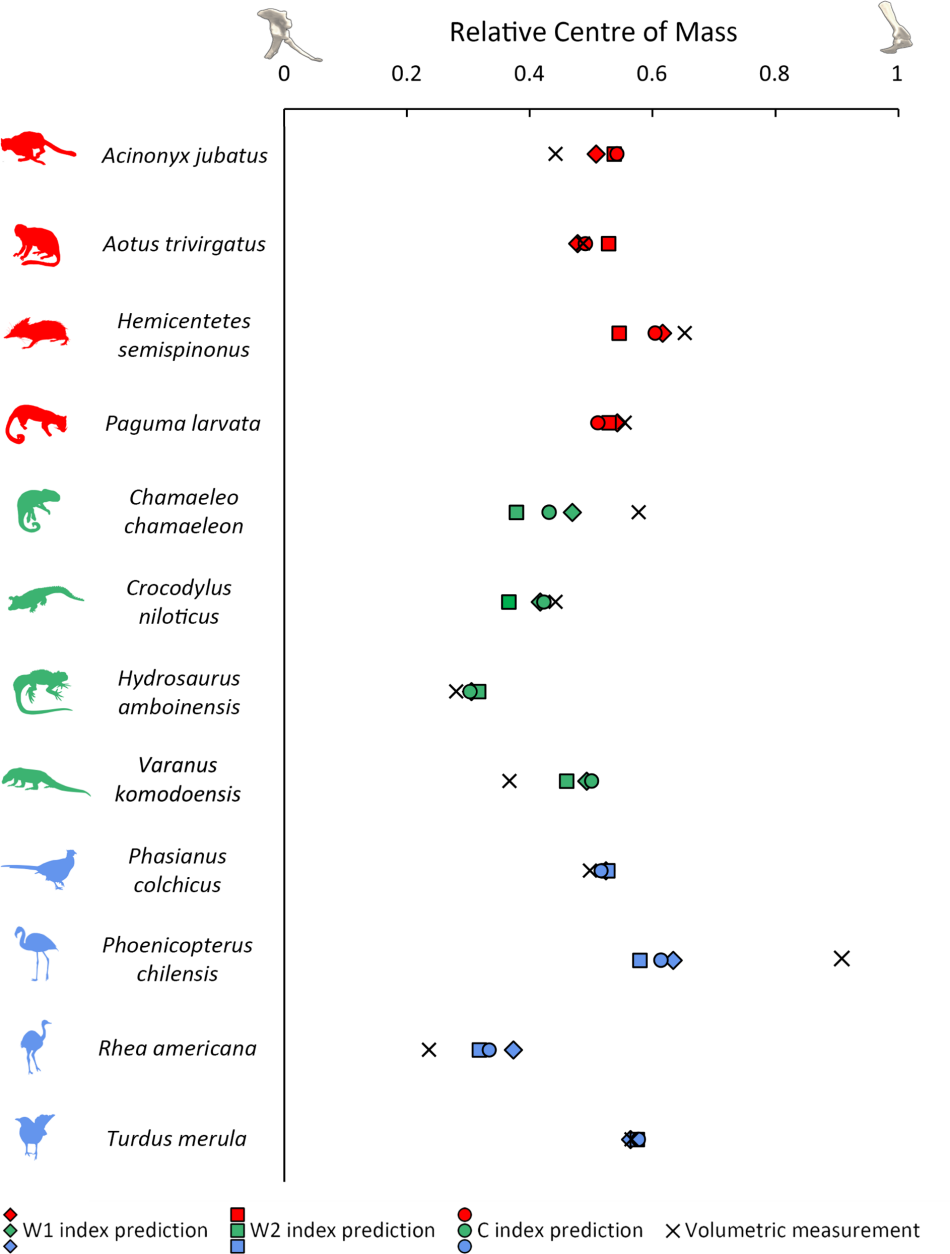
Differences between stylopodial index predictions and volumetric measurements of relative centre of mass in a selection of extant tetrapods. 0 = position of acetabulum and 1 = position of glenoid. Red = mammals, green = non-avian sauropsids, blue = birds.

In the mammal subset, OLS regressions provide support for hypothesis 1, as significant positive relationships (slope *p* < 0.05) between relative centre of mass and the W1 and C indices are recovered (figure 2; equations (3.1) and (3.2); electronic supplementary material, SM3). OLS model fit was found to be considerably greater for the W1 index (adjusted *R*^2^ = 0.709, AiCc = −36.8) than the C index (adjusted *R*^2^ = 0.383, AiCc = −29.3). Relative centre of mass prediction errors are lower for the mammal models than the other subsets, with mean differences of less than 5% glenoacetabular distance between measured and index-predicted relative centre of mass. When all mammals are considered, PGLS does not improve model fit relative to OLS, recovering no significant relationships between the stylopodial indices and relative centre of mass (electronic supplementary material, SM3). However, our boxplot analyses recover the cheetah (*Acinonyx jubatus*) as a residual outlier from all of the mammal humeral-to-femoral cross-sectional index OLS regressions, with relative centre of mass predictions that were more anterior than measured values by 7% to 11% glenoacetabular distance (figure 3; electronic supplementary material, SM3 and SM4). The subsequent exclusion of *A. jubatus* in an alternative set of mammal models considerably improves model fit, recovering significant positive relationships for all stylopodial indices across both the OLS and PGLS models, each of which had similar AiCc values and consistently low mean prediction errors of 1% to 3% glenoacetabular distance (figure 2*b*; equation (3.1); electronic supplementary material, SM3), with the OLS models exhibiting similarly high adjusted coefficients of determination (W1 index adjusted *R*^2^ = 0.899, W2 index adjusted *R*^2^ = 0.699, C index adjusted *R*^2^ = 0.818).

In the non-avian sauropsid subset, OLS regressions also provide support for hypothesis 1, as significant positive relationships between relative centre of mass and the W1 index (adjusted *R*^2^ = 0.397, AiCc = −25.1) and C index (adjusted *R*^2^ = 0.371, AiCc = −24.4) are recovered (figure 2; equations (3.3), (3.4); electronic supplementary material, SM3). The slope coefficients in the non-avian sauropsids provide evidence for distinct stylopodial index scaling trajectories from mammals and birds (i.e. steeper slope values), providing support for hypothesis 2 (figure 2; equations (3.3), (3.4); electronic supplementary material, SM3). PGLS mostly does not improve model fits for the non-avian sauropsids relative to OLS, only finding a significant relationship between relative centre of mass and the W2 index (electronic supplementary material, SM3). Overall prediction errors are higher in non-avian sauropsids than mammals, and similar to birds, with mean differences of 6% to 8% glenoacetabular distance between measured and index-predicted relative centre of mass. While not a residual outlier, each of the cross-sectional stylopodial index predictions of relative centre of mass in the common chameleon (*Chamaeleo chamaeleon*, which is the most front-heavy non-avian sauropsid in the dataset) are notable for being considerably more posterior than measured values by 12% to 20% glenoacetabular distance (figure 3; electronic supplementary material, SM4). We also recover significant positive relationships between the L index and relative centre of mass in non-avian sauropsids (electronic supplementary material, SM1 and SM3, figure S3), with support values indicating similar (albeit slightly weaker) model fit than the significant cross-sectional shaft indices (adjusted *R*^2^ = 0.353, AiCc = −24.0).

In birds, OLS regressions provide further support for hypothesis 1, as significant positive relationships between relative centre of mass and all humeral-to-femoral cross-sectional stylopodial indices are recovered, with slightly greater model fit for the C index (adjusted *R*^2^ = 0.675, AiCc = −67.6) than the W1 (adjusted *R*^2^ = 0.643, AiCc = −64.6) and W2 indices (adjusted *R*^2^ = 0.637, AiCc = −64.1) (figure 2; equations (3.5)–(3.7); electronic supplementary material, SM3). Like the mammals, the bird slope coefficients are considerably less steep than the non-avian sauropsids (figure 2; equations (3.4)–(3.7); electronic supplementary material, SM3), providing further evidence of distinct stylopodial scaling between different tetrapod clades, and thus adding support to hypothesis 2. Bird PGLS models received slightly lower AiCc values than the OLS models, but with only minor differences between mean relative centre of mass prediction errors (electronic supplementary material, SM3). The Chilean flamingo (*Phoenicopterus chilensis*) and common kingfisher (*Alcedo atthis*) are notably found to have very large prediction errors in each of the cross-sectional stylopodial index models, with predictions of centre of mass more anterior than measured values by 27−33% and 21−33%, respectively (electronic supplementary material, SM4). Despite these large residuals in absolute values, it is notable that predicted relative centre of mass differences between *P. chilensis*, *A. atthis* and many other birds remain consistent (figure 3; electronic supplementary material, SM4). When the ratites were excluded (figure 2*c*; electronic supplementary material, SM3), OLS regressions recover considerably smaller adjusted coefficients of determination (W1 index adjusted *R*^2^ = 0.289, W2 index adjusted *R*^2^ = 0.138, C index adjusted *R*^2^ = 0.321) than those including ratites (as expected when variance is reduced) but result in mostly minor changes to the overall linear trends. Furthermore, the 95% confidence intervals of each slope strongly overlap, and mean prediction errors remained consistent. When ratites were excluded, PGLS still recovered significant positive relationships between relative centre of mass and the W1 and C indices, but not the W2 index (electronic supplementary material, SM3). We also recover significant positive relationships between the L index and relative centre of mass in birds (electronic supplementary material, SM1 and SM3, figure S3). However, the support values for these relationships were weaker than those for the significant cross-sectional humeral-to-femoral index regressions (when all birds are included, L index adjusted *R*^2^ = 0.293, and AiCc = −43.5). The PGLS model also recovers positive relationships in between the L index and relative centre of mass in birds, but only when ratites are excluded (electronic supplementary material, SM1 and SM3, figure S3).

### 3.2. Coupling between mass-relative stylopodial dimensions and centre of mass in extant tetrapods

Significant positive relationships are recovered between relative centre of mass and mass-relative humeral cross-sectional shaft dimensions across multiple extant tetrapod subsets, providing support for hypothesis 3. When all mammals were considered, OLS regressions did not recover significant positive relationships between relative centre of mass and mass-relative stylopodial dimensions (electronic supplementary material, SM1 and SM3, figure S4). However, because *A. jubatus* is identified as an outlier in the initial regressions used to test hypotheses 1 and 2 (see above), to contextualize this prior result we also produced an alternative set of mass-relative stylopodial dimension regressions in which this taxon was also excluded. The exclusion of *A. jubatus* results in significant positive OLS and PGLS relationships between relative centre of mass and HW2/$\sqrt[{3}]{mass}$, as well as significant positive OLS relationships between relative centre of mass and HC/$\sqrt[{3}]{body\;mass}$ (electronic supplementary material, SM1 and SM3, figure S5). No significant relationships are found between relative centre of mass and the mass-relative dimensions of the femur in mammals. Significant positive OLS and PGLS relationships were recovered between relative centre of mass and HW2/$\sqrt[{3}]{body\;mass}$ in non-avian sauropsids (electronic supplementary material, SM1 and SM3, figure S4). Across birds, OLS and PGLS regressions recover significant relationships between relative centre of mass and all humeral shaft dimensions relative to $\sqrt[{3}]{body\;mass}$ (electronic supplementary material, SM1 and SM3, figure S4). When ratites are excluded, OLS relationships were significant for HW1/$\sqrt[{3}]{mass}$ and HC/$\sqrt[{3}]{mass}$ (electronic supplementary material, SM1 and SM3, figure S6). OLS regressions also recover significant relationships between relative centre of mass and all mass-relative femoral shaft dimensions across birds, but not when ratites are excluded (electronic supplementary material, SM1 and SM3, figures S4, S6).

In mammals, initial regressions did not recover significant relationships between mass-relative limb bone lengths and centre of mass (electronic supplementary material, SM1 and SM3, figure S7) However, the mouse (*Mus musculus*) and the lowland streaked tenrec (*Hemicentetes semispinosus*) were each found to be residual outliers from the FL/$\sqrt[{3}]{mass}$ OLS regressions (electronic supplementary material, SM3). Subsequent exclusion of these taxa resulted in both OLS and PGLS regressions recovering significant negative relationships between relative centre of mass and FL/$\sqrt[{3}]{mass}$ (electronic supplementary material, SM1 and SM3, figure S8). The OLS regressions did not recover significant relationships between mass-relative limb bone lengths and centre of mass in non-avian sauropsids, but significant positive slopes were recovered by PGLS for both the humerus and femur (electronic supplementary material, SM1 and SM3, figure S7). Both OLS and PGLS regressions also recovered significant positive relationships between centre of mass and HL/$\sqrt[{3}]{mass}$ in birds, irrespective of whether or not ratites were included (electronic supplementary material, SM1 and SM3, figure S8).

### 3.3. ‘Limb intrinsic’ stylopodial robusticity and centre of mass

In mammals, initial OLS regressions support some relationships between the increased intrinsic robusticity of the humerus (specifically, HW2/HL and HC/HL) and a more anterior relative centre of mass (electronic supplementary material, SM1 and SM3, figure S9). *M. musculus* is a residual outlier in the HW1/HL model, with its subsequent exclusion leading to a significant positive slope (electronic supplementary material, SM1 and SM3, figure S10). In non-avian sauropsids and birds, there are virtually no significant relationships between the intrinsic robusticity of the humerus and relative centre of mass (electronic supplementary material, SM1 and SM3, figure S9). One exception is a significant OLS negative relationship between HW2/HL and relative centre of mass in birds when ratites are excluded (electronic supplementary material, SM1 and SM3, figure S11), albeit with low support values (e.g. adjusted *R*^2^ = 0.11). The OLS regressions did not find significant relationships between limb-intrinsic femoral robusticity and centre of mass in any of the extant tetrapod clades. No significant relationships between limb-intrinsic robusticity and centre of mass are found when adopting PGLS models (electronic supplementary material, SM3). Overall, the lack of clear relationships between limb intrinsic robusticities and centre in multiple tetrapod clades emphasize the dimensions of the humerus relative to the femur as more suitable predictive proxies for fore–aft mass distribution than the intrinsic robusticity of any single limb bone, reinforcing hypothesis 1.

### 3.4. Application of extant linear models to dinosaur stylopodial indices

The following equations derived from the extant tetrapod OLS humeral-to-femoral cross-sectional stylopodial index models with significant slopes (*p* < 0.05) were applied to the non-avian dinosaur stylopodial indices.

*Mammal W1 index model:*


(3.1) $$\begin{array}{c} log10\left(relative\;centre\;of\;mass\right)=\;0.866*\;log10\left(W1\;index\right)-0.294\\ Mean\;relative\;centre\;of\;mass\;error=\;\pm 0.0198 \end{array}$$

*Mammal C index model:*


(3.2) $$\begin{array}{c} log10\left(relative\;centre\;of\;mass\right)=\;0.519*\;log10\left(C\;index\right)-0.271\\ Mean\;relative\;centre\;of\;mass\;error=\;\pm 0.0288 \end{array}$$

*Non-avian sauropsid W1 index model:*


(3.3) $$\begin{array}{c} log10\left(relative\;centre\;of\;mass\right)=\;1.240*\;log10\left(W1\;index\right)-0.355\\ Mean\;relative\;centre\;of\;mass\;error=\;\pm 0.0618 \end{array}$$

*Non-avian sauropsid C index model:*


(3.4) $$\begin{array}{c} log10\left(relative\;centre\;of\;mass\right)=\;1.375*\;log10\left(C\;index\right)-0.374\\ Mean\;relative\;centre\;of\;mass\;error=\;\pm 0.0621 \end{array}$$

*Bird W1 index model:*


(3.5) $$\begin{array}{c} log10\left(relative\;centre\;of\;mass\right)=\;0.656*\;log10\left(W1\;index\right)-0.229\\ Mean\;relative\;centre\;of\;mass\;error=\;\pm 0.0757 \end{array}$$

*Bird W2 index model:*


(3.6) $$\begin{array}{c} log10\left(relative\;centre\;of\;mass\right)=\;0.609*\;log10\left(W2\;index\right)-0.257\\ Mean\;relative\;centre\;of\;mass\;error=\;\pm 0.0843 \end{array}$$

*Bird C index model:*


(3.7) $$\begin{array}{c} log10\left(relative\;centre\;of\;mass\right)=\;0.652*\;log10\left(C\;index\right)-0.245\\ Mean\;relative\;centre\;of\;mass\;error=\;\pm 0.0750 \end{array}$$

**Figure 4.**
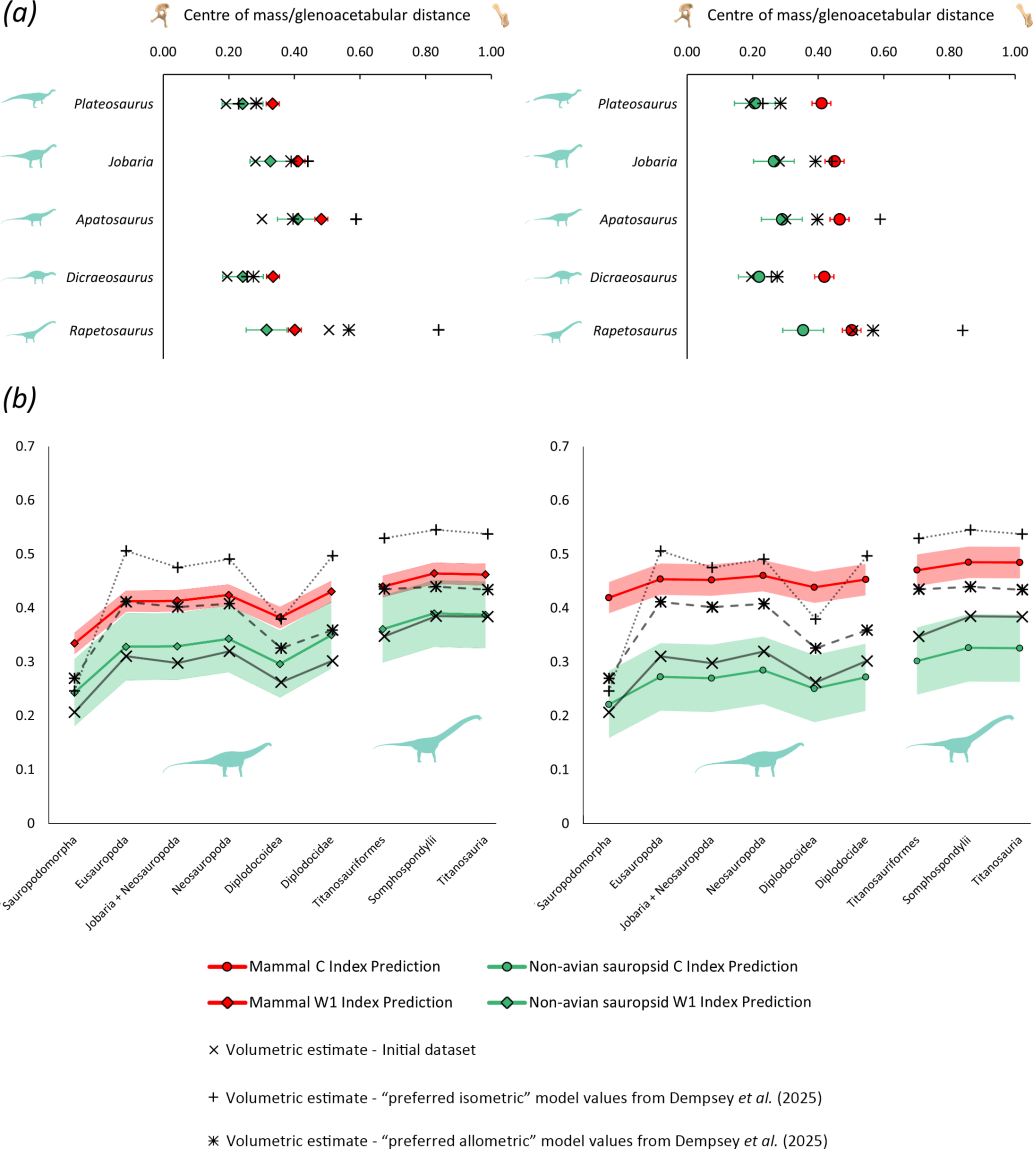
(*a*) Comparisons of extant quadruped index-estimated centres of mass in a selection of sauropodomorph dinosaurs with volumetric models. (*b*) Ancestral state reconstructions at major internal nodes in Sauropodomorpha, comparing the overall centre of mass evolutionary trajectories estimated by extant quadruped index equations (left = W1 index, right = C index) with those estimated from previous volumetric models. Shaded regions are derived from the mean relative centre of mass prediction errors of the extant index equations. Mammal-based and non-avian sauropsid-based estimates are focused on here based on assumptions of mechanical and functional similarity (i.e. quadrupedality). Bird-based estimates are provided in electronic supplementary material, SM7. The initial dataset primarily consists of the ‘spline-corrected’ model values from Bishop *et al*. [4].

Complete results of the extant equation applications are provided in electronic supplementary material, SM5 and SM6, with overall major trends summarized here. The alternative *A. jubatus*-excluded and ratite-excluded equations were also applied to the non-avian dinosaur stylopodial indices, producing similar results to the corresponding primary mammal and bird equations (electronic supplementary material, SM1 and SM5, tables S2, S3). Our comparisons herein focus on the extant-based C and W1 index equations, as these were the only indices found to have a significant relationship with relative centre of mass across all extant tetrapod clades. These equations result in centre of mass predictions that followed similar relative trends to previous volumetric estimates (figure 4; electronic supplementary material, SM1, table S2, figures S12–S13). The extant C index equations produce centre of mass estimates with a greater ranked similarity to the volumetric estimates (Spearman’s Rho = 0.70) than either width index (Spearman’s Rho = 0.60−0.61), irrespective of which equation subset is used (electronic supplementary material, SM1, table S2). In sauropodomorphs and ornithischians, centre of mass ancestral state reconstructions derived from the stylopodial index predictions follow similar relative patterns across major lineages to those derived from the volumetric estimates (figure 4; electronic supplementary material, SM1 and SM6, figure S12). Theropod centre of mass ancestral state reconstructions also follow similar relative trajectories between estimation methods (electronic supplementary material, SM6). However, due to theropods being represented by a smaller sample with narrower disparity in both centre of mass and limb bone dimensions, sauropodomorphs and ornithischians are focused on here. In sauropodomorphs, both index-based and volumetric centre of mass estimates each show an initial anterior shift in centre of mass between the base of Sauropodomorpha and Eusauropoda, a posterior reversion in centre of mass in Diplodocoidea from the base of Neosauropoda, and titanosauriform macronarians shifting towards more anterior centres of mass than diplodocoids (figure 4*b*).

In ornithischians, both the stylopodial and volumetric centre of mass estimates suggest an anterior shift in centre of mass between the base of Thyreophora and Ankylosauria, as well as between the base of Ceratopsia and Ceratopsidae, whereas centre of mass estimates at the major ornithopod nodes were less variable (electronic supplementary material, SM1, figure S12). Major differences in the reconstructed centre of mass evolutionary trajectories are identified at the Eurypoda and Neoceratopsia nodes, at which the index-estimated centres of mass were found to be respectively more anterior and more posterior relative to other nodes than in the volumetric estimates (electronic supplementary material, SM1, figure S12).

Despite these overall relative similarities, absolute differences are still found between volumetric estimates and index-based predictions of relative centre of mass. In general, the non-avian sauropsid index equations, particularly the C index, generate more posterior relative centre of mass estimates in dinosaurs than the mammal and bird equations (figure 4; electronic supplementary material, SM1 and SM5, figure S12). Overall, the non-avian sauropsid equations are also the most quantitatively consistent with the primary dataset of previous volumetric models, producing relative centre of mass estimates for each major dinosaur clade with an average difference of 5% to 9% glenoacetabular distance from models, compared with 6% to 21% when using the mammal equations, and 10% to 20% using the bird equations (figure 4*a*; electronic supplementary material, SM1, table S3, figures S12, S13). In most dinosaurs, the bird and mammal index equations predict relative centres of mass that were more anterior than the primary dataset of previous volumetric estimates, only closely aligning with them in taxa with extreme front-heavy morphologies, such as the longest necked sauropods (e.g. *Rapetosaurus*, figure 4*a*), large-headed ceratopsians, taxa with proportionally long torsos (e.g. *Dilophosaurus*), or taxa with relatively small stylopodial indices (e.g. *Tyrannosaurus*) (electronic supplementary material, SM1, figure S13). Most extant-based stylopodial index predictions of centre of mass in *Stegosaurus* and *Tenontosauru*s are notable for being considerably more anterior than the previous volumetric estimates (electronic supplementary material, SM1 and SM5, figure S13). Conversely, most extant-based stylopodial index predictions for *Protoceratops* are considerably more posterior than the previous volumetric centre of mass estimate (electronic supplementary material, SM1 and SM5, figure S13). These differences in taxon-specific estimates underpin the major differences between the ancestral state reconstructions of centre of mass (i.e. a more anterior centre of mass at the base of Eurypoda, and a more posterior centre of mass at the base of Neoceratopsia; electronic supplementary material, SM1, figure S12). Our analyses of the Dempsey *et al*. [31] sauropodomorphs demonstrate that relative trajectories in centre of mass evolution derived from different volumetric modelling methods remain consistent with each other and with extant stylopodial index-based estimates (figure 4). However, the absolute similarity between these estimates may considerably vary (figure 4). The more posterior sauropodomorph centre of mass estimates from the initial dataset, which were mostly derived from the Bishop *et al*. [4] ‘spline-corrected’ scaling factor applied to previous convex hull models (see §4.4), were closely comparable with the results of the non-avian sauropsid stylopodial C index equations, whereas centre of mass estimates derived from the Dempsey *et al*. [31] convex hull models were considerably more anterior, with some skewing closer to or even exceeding the mammal stylopodial index equations (figure 4).

### 3.5. Dinosaur linear regressions

**Figure 5.**
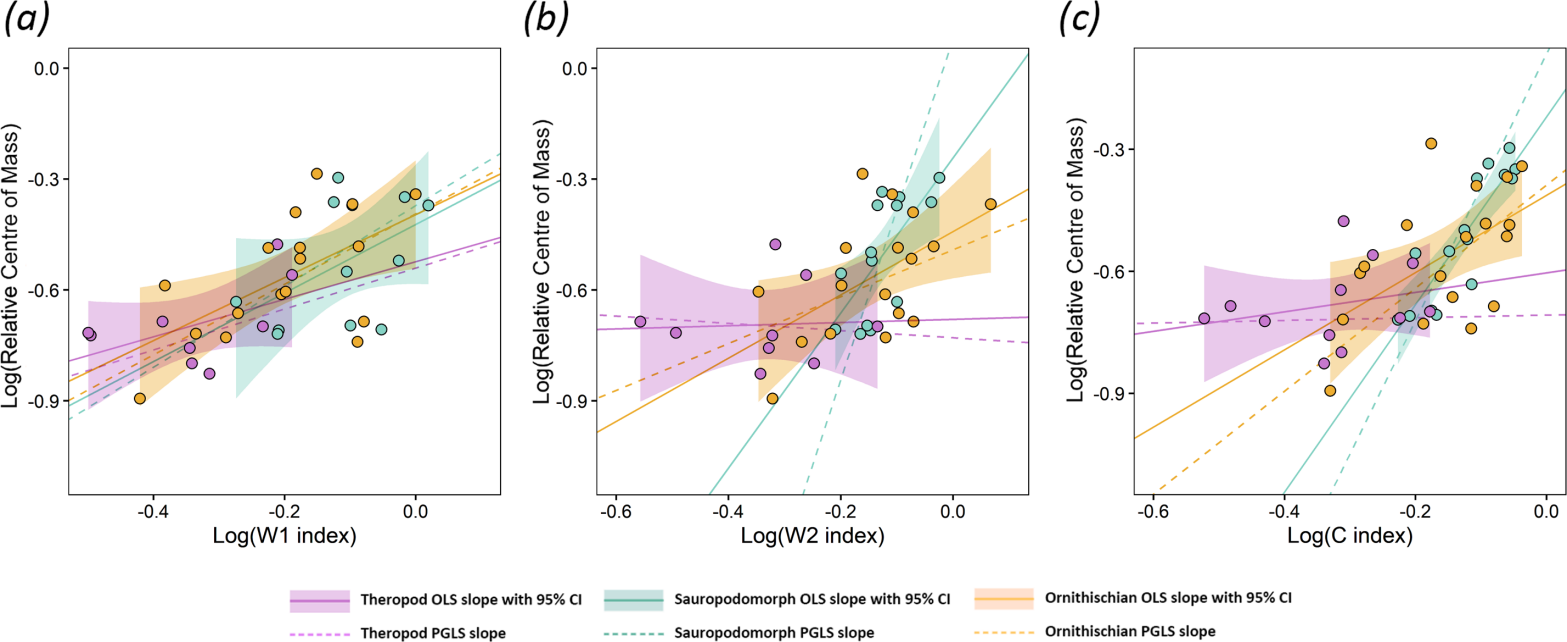
Scatter plots showing the results of the volumetric estimates of relative centre of mass–stylopodial index regressions in non-avian dinosaurs. (*a*) W1 index, (*b*) W2 index, (*c*) C index, as described in the text.

Complete data from the dinosaur linear regressions are provided in electronic supplementary material, SM7, with major overall trends summarized here. Significant positive OLS relationships are most notably recovered between previous volumetric relative centre of mass estimates and cross-sectional stylopodial indices in sauropodomorphs when using the C index (figure 5; electronic supplementary material, SM7), with a low mean centre of mass prediction error of 4% glenoacetabular distance (figure 5; electronic supplementary material, SM5). A significant relationship is also found between centre of mass and the W2 index of sauropods (figure 5; electronic supplementary material, SM7). Notably, support values (C index adjusted *R*^2^ = 0.759, W2 index adjusted *R*^2^ = 0.429) indicate considerably stronger relationships between relative centre of mass and each of these indices in sauropodomorphs than those found in other dinosaur groups. A significant relationship is also noted between relative centre of mass and the W1 index in sauropodomorphs, but only when phylogeny was incorporated via PGLS. Significant positive relationships are also found between the L index and previous volumetric relative centre of mass estimates in sauropodomorphs, albeit with lower support values (OLS adjusted *R*^2^ = 0.438) than or greater mean centre of mass prediction errors (8–12%) than the C and W2 cross-sectional indices (electronic supplementary material, SM1 and SM7, figure S14). Adopting a PGLS model does not broadly improve sauropodomorph model fits, mostly producing higher AiCc values in the cross-sectional stylopodial index regressions, and either only slightly changing or increasing mean prediction errors (electronic supplementary material, SM7). In ornithischians, OLS regressions also recover significant positive relationships between previous volumetric relative centre of mass estimates and all the cross-sectional humeral-to-femoral indices, with each receiving similar support values and comparable mean prediction errors (~7% glenoacetabular distance; figure 5; electronic supplementary material, SM7). Incorporation of phylogeny into the ornithischian linear models via PGLS does not improve model fit. Notably, the ornithischian cross-sectional index models all estimate a more posterior relative centre of mass in *Protoceratops* than previous volumetric estimates (>20% glenoacetabular distance; electronic supplementary material, SM8), mirroring differences between extant index-predicted centres of mass and previous volumetric models (electronic supplementary material, SM1 and SM7, figure S13). Significant relationships were not recovered between stylopodial indices and relative centre of mass in theropods (electronic supplementary material, SM7). However, despite the lack of slope significance, mean prediction errors of the theropod slope equations were still relatively low (<4%), reflecting the fact that theropod centres of mass are overall more posteriorly restricted than in the other dinosaur clades (figure 5; electronic supplementary material, SM7).

No significant relationships are recovered between the intrinsic robusticity of the humerus and previous volumetric estimates of centre of mass in non-avian dinosaurs (electronic supplementary material, SM1 and SM7, figure S15). Significant negative relationships are found between FW1/FL and relative centre of mass estimates in sauropodomorphs, albeit with lower support values than the significant humeral-to-femoral index models (electronic supplementary material, SM7). Significant negative relationships (albeit with broad residual spreads) between relative centre of mass estimates and FW1/FL in ornithischians and FC/FL in sauropodomorphs are recovered by PGLS, but not by the corresponding OLS models (electronic supplementary material, SM1 and SM7, figure S15).

The regression models derived from the alternative Dempsey *et al*. [31] sauropodomorph centres of mass estimates mirror those derived from the initial volumetric dataset, showing significant positive relationships between relative centre of mass and most of the same stylopodial indices (electronic supplementary material, SM1 and SM7, figures S17–S22). When both recover significant slopes, the ‘preferred allometric’ centre of mass regressions show stronger support values than the ‘preferred isometric’ centre of mass regressions (i.e. higher adjusted coefficients of determination and lower mean prediction errors; electronic supplementary material, SM7). The overall poorer fit of the ‘preferred isometric’ regressions is partially driven by the much wider total range of centres of mass in this model set (electronic supplementary material, SM8), resulting in much larger prediction errors for these taxa than both the corresponding ‘preferred allometric’ predictions, particularly in the most extremely proportioned taxa (e.g. in *Mamechisaurus*, the W2 OLS index model predicts a centre of mass 40% glenoacetabular distance more posterior than the volumetric estimate). *Neuquensaurus* is also found to be a major residual outlier from the alternative C index models, as well as the W1 index and FW1/FL model when using the Dempsey *et al*. [31] ‘preferred isometric’ centres of mass (note that humeral length was not available for the *Neuquensaurus* specimens from which cross-sectional dimensions were measured, and it is thus not included in the intrinsic humeral robusticity models). These particularly large residuals partially result from *Neuquensaurus* being reconstructed with proportionally very large tail volumes by Dempsey *et al*. [31], particularly in the ‘preferred isometric’ models, which leads to more extreme rear-heavy centre of mass estimates than sauropods with similar relative limb dimensions (electronic supplementary material, SM2, SM7). Subsequent exclusion of *Neuquensaurus* improved model support values (electronic supplementary material, SM1 and SM7, figures S21, S22).

## 4. Discussion

### 4.1. Overarching trends in relative stylopodial shaft robusticity in quadrupedal tetrapods

Overall, our results indicate coupling between body mass distribution (i.e. centre of mass) and fore-to-hind limb stylopodial robusticity across the extant tetrapod subsets explored herein. Specifically, the linear regressions show strong support for our first overarching hypothesis that proportionally greater anterior loading on tetrapod limbs in association with a more front-heavy body is coupled with relatively more robust forelimbs to compensate (hypothesis 1). Significant relationships between fore-to-hind limb stylopodial shaft robusticity and relative centre of mass are found across all extant tetrapod subsets examined in this study, and are most apparent in the mammal subset, especially when shaft robusticity is measured parallel to the planes of elbow and knee extension (the W1 index, figure 2).

While relationships between relative centre of mass and cross-sectional humeral-to-femoral stylopodial indices were found to be significant in both mammals and non-avian sauropsids, the scaling exponents and overall strength of the support values are distinct between each group (figure 2; equations (3.1)–(3.4); electronic supplementary material, SM3). We interpret this variation as support for our second overarching hypothesis, which proposed that the existence of these differences is related to postural disparity (hypothesis 2; figure 1*a*). All quadrupedal tetrapod stylopodia experience axial compression and bending across their midshafts, but those of sprawling taxa are subjected to higher degrees of torsion than those of parasagittal taxa [16]. The dominance of axial loading in parasagittal mammal limbs may explain why relative increases in limb bone shaft thickness occur more predictably along the anteroposterior (W1) anatomical axis than in non-avian sauropsids, in which limb bone loading regimes may be more complex and variable. The non-avian sauropsids sampled here exhibit a greater spread from overall trendlines than the mammals (figure 2), indicating a broader range of relative limb bone dimensions and body shapes, reflecting adaptations to a more complex suite of locomotor behaviours across the group (e.g. high walking in crocodylians, facultative bipedality in some lizards). These distinct and varied loading regimes may also be associated with higher limb bone safety factors in sprawling tetrapods than in mammals [17,23], further explaining differences in the scaling exponents of their stylopodial dimensions.

Our results also suggest that in most tetrapods, relative fore–aft mass distribution is more strongly associated with the cross-sectional dimensions of the humerus relative to the femur than with the intrinsic robusticity of any singular limb bone (i.e. its cross-sectional dimensions relative to its length) or relative humeral-to-femoral length. Variation in limb bone length affects the way in which they are loaded. For example, limb bone length, alongside cross-sectional dimensions, is proportional to the bending loads experienced during locomotion [42,43]. While variation in gait, posture, and the resultant loading regimes incurred upon the limb are thus theoretically associated with limb length, our centre of mass scaling data suggest that neither limb bone length, nor the robusticity of the shaft relative to said length, are consistently adaptively coupled with the total proportion of body mass supported by each limb across tetrapods as a whole, at least in the present dataset. Mammals may be an exception, as our regressions tentatively suggest that front-heavy taxa trend towards thicker humeral shafts relative to their length (albeit with weaker support values than other indices). Ultimately, the lack of clear universal relationships between centre of mass, limb bone lengths and limb-intrinsic (length-relative) shaft thickness is an intuitive expectation when viewed in the context of previous work. Campione & Evans [25] previously demonstrated that the cross-sectional dimensions of limb bone shafts are more statistically robust proxies for total mass than limb bone lengths. Accordingly, one would expect that those same limb bone shaft dimensions to also closely reflect the spatial distribution of said mass. While limb segment lengths are ultimately still expected to vary with body mass properties, this variation may also be complicated by a suite of other factors related to posture, locomotor performance and other behavioural traits [44] in addition to the loading imposed by body weight alone.

While our results provide support for differential scaling between limb bone cross-sectional dimensions and fore–aft mass distribution between tetrapod clades, it should be acknowledged that our mammal dataset is presently small (*n* = 10), being limited to rodents, primates and carnivorans. It is therefore unclear if the reasonably well-constrained trends recovered here are consistent across all mammals (especially larger taxa absent from our dataset), or if a broad spectrum of variation similar to that recovered in the non-avian sauropsids should instead be expected. For example, the recovery of *A. jubatus* as a residual outlier across most of the initial humeral-to-femoral cross-sectional index models suggests that specific or ‘extreme’ functional adaptations may lead to deviations in stylopodial dimensions and body proportions. In the case of *A. jubatus*, greater peak stresses during high-performance running are associated with forelimb skeletal adaptations distinct from other carnivorans [45], which, as shown here, appear to include relatively more robust humeri than expected for its centre of mass, at least compared with the other mammals in the dataset. *M. musculus* is found to be a residual outlier in both the HW1/HL and HL/$\sqrt[{3}]{mass}$ models. This proportional distinctiveness may reflect behavioural or functional differences from other mammals, but may also be related to differences in absolute size (the body mass of *M. musculus* is two orders of magnitude smaller than that of the majority of the other mammals studied herein). While not necessarily residual outliers from overall patterns, other mammals included in our dataset may further represent functional extremes. For example, the lowland streaked tenrec (*Hemicentetes semispinosus*), which is the most front-heavy mammal in our dataset, has particularly thick humeral shafts, potentially reflecting the use of the forelimbs in burrowing behaviours [46]. Careful consideration of morphologically or behaviourally distinct taxa is thus important when assessing the scaling trends discussed herein. It is also possible that a more broadly sampled dataset encompassing a greater range of mammalian morphologies and behaviours might not recover these ‘extreme’ taxa as outliers.

Functional and ecological factors underpinning variable morphologies might also explain the largest residuals and prediction errors seen in non-avian sauropsids. For example, despite not being a residual outlier, relative centre of mass in the Komodo dragon (*Varanus komodoensis*) is predicted by both the W1 index and C index models to be more anterior than many smaller lizards, considerably contrasting with the volumetric models, which reconstruct a relatively posterior centre of mass in *V. komodoensis* (figure 3). This discrepancy is due to the proportionally very robust humerus of *V. komodoensis*, which has a considerably greater C index (1.13) than other lizards (0.78−1.06), as well as a high W1 index (1.09). The black caiman (*Melanosuchus niger*) is also estimated from its stylopodial dimensions to be considerably more front-heavy than volumetric measurements. Perhaps these discrepancies result from larger lizards and crocodylians experiencing greater peak stresses, distinct loading or postural distinctiveness of the forelimb in association with their increased body size (64 kg in our studied *V. komodoensis* specimen, 90 kg in our studied *M. niger* specimen), leading to relatively robust humeri for their centres of mass. In the common chameleon (*Chamaeleo chamaeleon*), centres of mass estimated from the humeral-to-femoral cross-sectional stylopodial indices are notably more posterior than the volumetrically measured values (figure 3), indicating a more gracile humerus than would be predicted based on the overall trendline. This relative gracility may be associated with the partially parasagittal gait of chameleons during arboreal movement [47], as parasagittal posture has previously been associated with lower limb bone safety factors than a sprawling gait [17]. Going forward, verification of these speculations, and quantifications of any associations between limb bone size, centre of mass, posture and ecology would require an increased sampling of taxa (particularly larger species) as well as detailed comparisons to *in vivo* loading and kinematic data.

### 4.2. Stylopodial shaft scaling in birds

Despite the fact that bird forelimbs are not subjected to the ground reaction forces experienced by quadrupeds, our results reveal that increases in the relative humeral-to-femoral shaft dimensions of birds are significantly and positively associated with the relative position of the centre of mass (figure 2; electronic supplementary material, SM3). This is consistent with the fact that the wings (and thus humeri) of birds are nonetheless subject to variable loading during flight. More aerially inclined (forelimb-dominated) birds (figure 1*a*) [48] require larger wings and flight muscles compared with infrequently aerial or flightless (hind limb-dominated) birds (figure 1*a*) [48], leading to anterior shifts to their centres of mass [7]. We demonstrate herein that these shifts in the relative centres of mass of birds are, as in quadrupedal tetrapods, coupled with increases in relative humeral-to-femoral robusticity, broadening the support for hypothesis 1. Support values for each recovered relationship between the humeral-to-femoral cross-sectional stylopodial indices and relative centres of mass in birds were similar (electronic supplementary material, SM3). This lack of clear preferential directionality in increased robusticity likely results from the circular and less eccentric stylopodial cross-sections seen in birds (e.g. figure 1*e*), which are better suited to resist the high torsional loads enacted on the humerus during flight (e.g. [16,17]). These results further highlight how differences in stylopodial index scaling exponents between different tetrapod groups are related to function, adding support to hypothesis 2.

It is also possible that wider residual spreads and differential scaling between centre of mass and limb bone dimensions in birds and other tetrapod groups result from differences in the internal morphology of the bones, and the resultant effect of these differences on how the limbs are loaded. The limb bones of birds generally have relatively thin cortical walls compared with other tetrapods [49]. Differences in both the density and more complex shapes and architecture of cortical and cancellous bone also influence the absolute mechanical performance of limb bones (e.g. [50,51]). However, external and internal limb bone dimensions appear to be scale-invariant within non-avian sauropsids and mammals [52], and internal and external shaft measurements are similarly robust as predictors of body mass in both birds and mammals [30]. It is therefore likely that external limb bone dimensions alone are still an effective proxy for differences in mass distribution and other mass properties, at least in relative terms, or within specific clades.

### 4.3. A more robust humerus, or a more gracile femur?

Given the strong relationship between limb bone shaft dimensions and body mass [25], it is intuitive to hypothesize that more anterior mass distribution in tetrapods would be coupled with the increased humeral shaft robusticity relative to body mass as well as relative to the femur, as an adaptation to greater total forelimb stresses (hypothesis 3). Our regressions between relative centre of mass and mass-relative humeral shaft dimensions show some tentative support for this hypothesis in mammals (when *A. jubatus* was excluded), non-avian sauropsids, and birds (electronic supplementary material, SM1, figures S6–S8, SM3)—although notably, the anatomical planes within which mass-relative humeral robusticity increases alongside centre of mass are not necessarily the same planes within which increases in relative humeral-to-femoral shaft robusticity occur. Overall, this reflects the functional importance of a larger humerus as a load bearer in front-heavy tetrapods, during both weight support and locomotion in quadrupedal tetrapods, and during sustained flight in forelimb-dominated birds. By contrast to the humerus, significant relationships were not recovered between mass-relative femoral shaft dimensions and centre of mass in either the mammals or the non-avian sauropsids. This suggests that changes in relative stylopodial dimensions coupled with anterior shifts in mass distribution are likely to be more driven by constraints enforced upon the humerus, rather than a relaxation of constraints (e.g. alleviated total hind limb stresses) on the femur. However, when ratites are included in the bird subset, weak coupling between a more posterior centre of mass and the robusticity of the femur relative to body mass is found (electronic supplementary material, SM1, figure S4, SM3). This pattern is consistent with the obligate terrestrial lifestyle of ratites, which likely leads to greater and more frequent femoral strain compared to volant birds, and evidently removes any load-bearing constraints from the wings, explaining the relatively more robust femora and gracile humeri.

### 4.4. Can evolutionary patterns in centre of mass be predicted from relative limb bone sizes?

The consistent significant associations between relative humeral-to-femoral stylopodial robusticity and centre of mass found across the extant tetrapods sampled here suggest the potential to predict mass distribution in wider arrays of tetrapods, including fossil taxa. Centre of mass estimates derived from large sample sizes of relatively simple to obtain skeletal measurements may therefore have the potential to underpin broad-scope macroevolutionary studies on body mass distribution across major tetrapod clades, allowing for further quantitative exploration of previously hypothesized associations between limb dimensions and aspects of whole-body morphology and locomotion [7,27,28]. However, unlike relationships between total body mass and stylopodial size, which have previously been shown to be similar between different tetrapod groups [25], the relationships between stylopodial indices and relative centres of mass differ between the subsets considered here. For example, the mammal, bird, and non-avian sauropsid slopes converge at anterior relative centres of mass and high W1 and C indices, but diverge negatively (figure 2). Such disparity indicates that the use of such relationships to estimate centre of mass in fossil taxa will require either *a priori* selection of the most appropriate predictive model, or acknowledgement of broad uncertainties if an appropriate model cannot be clearly identified (figure 4; electronic supplementary material, SM1, figures S12–S13). Our application of the extant stylopodial index equations to non-avian dinosaur data and the subsequent comparison of the results with previous volumetric models (figure 4; electronic supplementary material, SM1, SM6, SM7, figures S12–S13) acts as a case study from which these considerations can be explored further. Major relative differences in dinosaur centre of mass recovered by previous volumetric models were also recovered by the index equations, as shown by the high ranked similarity between estimates, particularly for the C index equations (Spearman’s Rho = 0.70), as well as the similar reconstructed trajectories in centre of mass evolution across major dinosaur lineages (figure 4; electronic supplementary material, SM1, figure S12). This general consistency suggests that the predictive equations presented here may have high utility in future studies of body shape macroevolution, as they may be able to accurately recover broad patterns in centre of mass disparity across large datasets of stylopodial indices, including taxa for which full body reconstructions are infeasible due to limited fossil remains. However, it is evident that extant-scaling prediction models will not necessarily generate precise absolute estimates of centres of mass (figure 4; electronic supplementary material, SM1, figures S12–S13). For example, the mammal and bird equations generate more anteriorly skewed centre of mass estimates in most dinosaurs than the non-avian sauropsid equations, which produce more posterior values closer to the primary set of previous volumetric estimates (figure 4; electronic supplementary material, SM1, figures S12–S13). The non-avian sauropsid equations may therefore represent a quantitatively ‘preferred’ model for future studies on non-avian dinosaurs, whereas large absolute differences between volumetrically estimated centres of mass in quadrupedal dinosaurs and mammal-based stylopodial equation predictions may reflect general differences in gross limb proportions and mechanics. Unlike mammals, quadrupedal dinosaurs evolved from bipedal ancestors [53,54], with many taxa variably retaining long, robust hind limbs that would have undoubtedly played a significant role in both weight bearing and propulsion [27,31,55–57]. These quantitative differences between equations may affect more focused interpretations of the anatomy and biomechanics of a specific taxon, and thus highlight the need for careful consideration of the varied applicability of extant-based scaling models to fossil taxa. However, the ability of extant-based equations to recover relative patterns in centre of mass between taxa that are consistent with previous volumetric estimates means that broader interpretations of wider evolutionary patterns will remain consistent in spite of absolute differences. In any case, caution should still be exercised when attempting to interpret potential coupling between centre of mass and limb bone dimensions in possible outlier taxa from otherwise consistent evolutionary trends.

The additional regressions of stylopodial indices against previous volumetric estimates of centre of mass in dinosaurs revealed differences in scaling exponents and residual spreads between each of the major clades (sauropodomorphs, theropods and ornithischians). As with the results from the extant regressions, this variation may be underpinned by differences in locomotor biomechanics between taxa. In sauropodomorphs, it is the C and W2 indices of the stylopodial shafts, not the W1 index, that were found to have the strongest associations with previous volumetric centre of mass estimates (figure 5; electronic supplementary material, SM5). This suggests that, unlike in extant quadrupedal tetrapods, adaptational responses to varied anteroposterior centre of mass position (and by inference relative anteroposterior loading) in sauropods were not achieved via increases in anteroposterior humeral shaft thickness. The increased mediolateral width of the humeral shafts relative to the femoral shafts in front-heavy titanosauriformes (e.g. *Rapetosaurus*) may also have been associated with their widely spaced postures [26], further highlighting their mechanical and proportional distinctiveness from extant parasagittal mammals, which typically adopt narrower-gauge postures. We also find some evidence for the femora of more rear-heavy sauropodomorphs being both longer relative to the humerus and intrinsically more robust (thicker shafts relative to their length). In early-diverging taxa such as *Plateosaurus*, this is likely reflective of the retention of the ancestral bipedal dinosaurian body plan, whereas in several more deeply nested quadrupedal sauropods exhibiting secondary posterior reversions in centre of mass (e.g. diplodocids), it may instead be associated with the additional loading incurred by their enlarged tails [31].

In ornithischians, evidence for plane-specific relative thickening of the humeral shafts as centre of mass becomes more anterior is also limited, with significant but unconstrained (i.e. high residual spread) relationships being recovered for each stylopodial index (figure 5; electronic supplementary material, SM7). The PGLS regressions also suggest that at least some front-heavy ornithischians trended towards less intrinsically robust femora in the W1 plane (i.e. narrower shafts relative to their length; electronic supplementary material, SM7). This wide spectrum of variation may reflect postural and mechanical disparity between different ornithischian clades, each of which evolved quadrupedality convergently [2,27,58]. Taxa such as ceratopsids and ankylosaurs are often hypothesized to have had relatively splayed gaits [58,59], and thus, as in extant sprawling taxa [16], may have experienced a more complex array of torsional loads across their limb bone shafts than taxa with narrower-gauge postures such as hadrosaurs [58,59]. Following our hypothesis that posture explains the different scaling exponents between stylopodial dimensions and centre of mass in extant tetrapods, well-constrained, whole-clade relationship between limb bone dimensions and centre of mass in a group with the apparent locomotor diversity of ornithischians would not necessarily be expected.

Extant birds are a radiation of theropods, yet our bird equations generally do not produce quantitatively similar centre of mass predictions to previous non-avian theropod volumetric reconstructions (electronic supplementary material, SM1, figure S13). Furthermore, the theropod linear regressions also recover little evidence of constrained relationships between relative limb bone cross dimensions and volumetrically reconstructed centres of mass. Ultimately, the lack such a relationship in theropods is expected, as the forelimbs of non-avian theropods are very varied in size and function [60] and, importantly, are not constrained by the mechanical demands of the flight-associated loads experienced by their avian counterparts.

While stylopodial dimensions appear to be an effective proxy for interpreting relative changes in fore–aft mass distribution and posture across broad evolutionary lineages of tetrapods, they cannot necessarily be used to predict the absolute mechanical performance of any given taxon. Holistic, quantitative modelling of the relationship between limb biomechanics and body shape in a single animal ultimately requires detailed considerations of multiple anatomical metrics, some of which are not always practically or non-destructively measurable in fossil taxa (e.g. detailed limb bone cortical architecture). These practical considerations underpin our focus on external limb bone measurements.

It must also be acknowledged that subjectivity and variation in the methods used to volumetrically reconstruct dinosaurs makes cross-validation of stylopodial index predictions complicated. Such complications are demonstrated by our sensitivity analysis of methodologically distinct centre of mass estimates in sauropodomorphs (figure 4). Ultimately, we find that major relative shifts in centre of mass between sauropodomorph clades estimated from methodologically distinct volumetric models are each consistent with those estimated from the extant-based stylopodial equations. However, the absolute values vary considerably, particularly in the more front-heavy clades such as titanosauriformes (figure 4*b*). This absolute variation reflects differences in the underlying methods from which volumetric centre of mass estimates may be derived. The Dempsey *et al*. [31] convex hull models were based on expansions of mathematically defined minimal 3D envelopes grounded in extant sauropsid soft tissue scaling data, whereas most of the sauropodomorph values collated in our initial dataset were derived from the Bishop *et al*. [4] ‘spline-corrected’ scaling factor, which was based on systematic differences between previous hull-based envelopes and hand-illustrated spline or slice-based soft tissue outlines [3,61,62], allowing for fairer comparison between models constructed via different methods. Multiple factors may explain the differences between these methodologically distinct centre of mass estimates, many of which, including subjective tendencies regarding the spatial distribution of soft tissue in hand-modelled outlines, have been quantified and discussed in several previous works (e.g. [1,35,63–65]). Differences in both appendicular and axial body posture may also influence reconstructed centres of mass in fossil animals. However, sensitivity analyses carried out by this study (electronic supplementary material, SM1.1) and others [3,31] on both extant and extinct tetrapods lead us to conclude that the influence of posture alone on relative anteroposterior mass distribution is likely to be fairly minor in all but the most extremely proportioned taxa. Ultimately, subjective modelling assumptions are important factors to consider when comparing index-derived centre of mass estimates in dinosaurs to previous volumetric models. However, a considerable body of work based on both fossil and extant animals has demonstrated that while absolute centre of mass estimates for individual taxa may vary considerably, major relative trends between them remain consistent between different modelling methods and/or assumptions (e.g. [1,3,7,31,66,67]). Further development of volumetric modelling methods, particularly those that ground body segment reconstructions in empirical data from extant animals [7,31,33], represent promising new avenues for further research beyond the scope of this particular study, and may produce a more systematic framework to which centres of mass predicted from stylopodial indices can be compared. However, the results presented herein nonetheless open up discussions about how limb bone dimensions may have adapted to varying mass distribution in dinosaurs, and how these relationships differed both within Dinosauria and relative to extant tetrapods. Furthermore, major differences between index predictions and both past and future volumetric estimates may provide a framework within which genuine biological variation from overall trends may be illuminated [32].

## 5. Conclusions

This study quantitatively associates changes in anteroposterior mass distribution in tetrapods with relative stylopodial robusticity, illuminating the adaptive development of humeral and femoral shafts according to the mechanical demands of stance and locomotion. Our results build upon previous findings that limb bone shaft dimensions are tied to both body mass properties and the regimes under which limbs are loaded. Specifically, our results emphasize that taxa with more anteriorly displaced centres of mass trend towards proportionally more robust humeri compared with the femur, which likely evolved to compensate for the greater relative loading of body mass onto the humerus. We also showed that the anatomical plane within which robusticity increases varies between different tetrapod groups, in a manner that is consistent with different loading regimes induced by disparate postures. In extant quadrupedal taxa, relative increases in fore-to-hind limb stylopodial shaft robusticity were most pronounced along the plane of elbow and knee extension. This pattern was most pervasive in the mammals studied herein, and is likely associated with the mitigation of greater parasagittal bending and compression forces than in non-avian sauropsids, which exhibit a more varied array of locomotor styles and therefore likely experience less directionally constrained limb bone loading. In birds, we found that increases in relative shaft robusticity are less constrained to specific anatomical planes, which we associated with the need to retain a relatively round cross section to resist torsional loads. In most tetrapod groups, we also find that relative humeral-to-femoral cross-sectional dimensions are a stronger predictive proxy of centre of mass than relative limb bone lengths or the cross-sectional measurements of individual limb bones relative to their length. Overall, these results indicate that predictive models could be applied to the stylopodial dimensions of much larger datasets of extant and extinct tetrapods to estimate their relative centres of mass, and to investigate broader evolutionary trends. However, future work should remain mindful of certain caveats. Differential scaling in extant taxa indicates the need for *a priori* model choice, or acceptance of broad uncertainties in absolute predicted values. While these sources of uncertainty should be acknowledged, the predictive models result in overall patterns in relative centre of mass variation across dinosaurs that are congruent with those estimated from volumetric models. Our linear equations therefore have the potential to estimate differences in relative mass distributions in more fragmentary fossil taxa for which full body models cannot be constructed, providing a framework to interpret tetrapod body plan evolution across deep time.

## Data Availability

Full anatomical data and a summary of their sources are included in electronic supplementary material, SM2. Newly prepared/edited 3D models used in this study are available at the following repository link: [68]. Previously published 3D models used in this study are available in repositories [69,70]. Supplementary material is available online [71].
